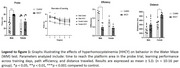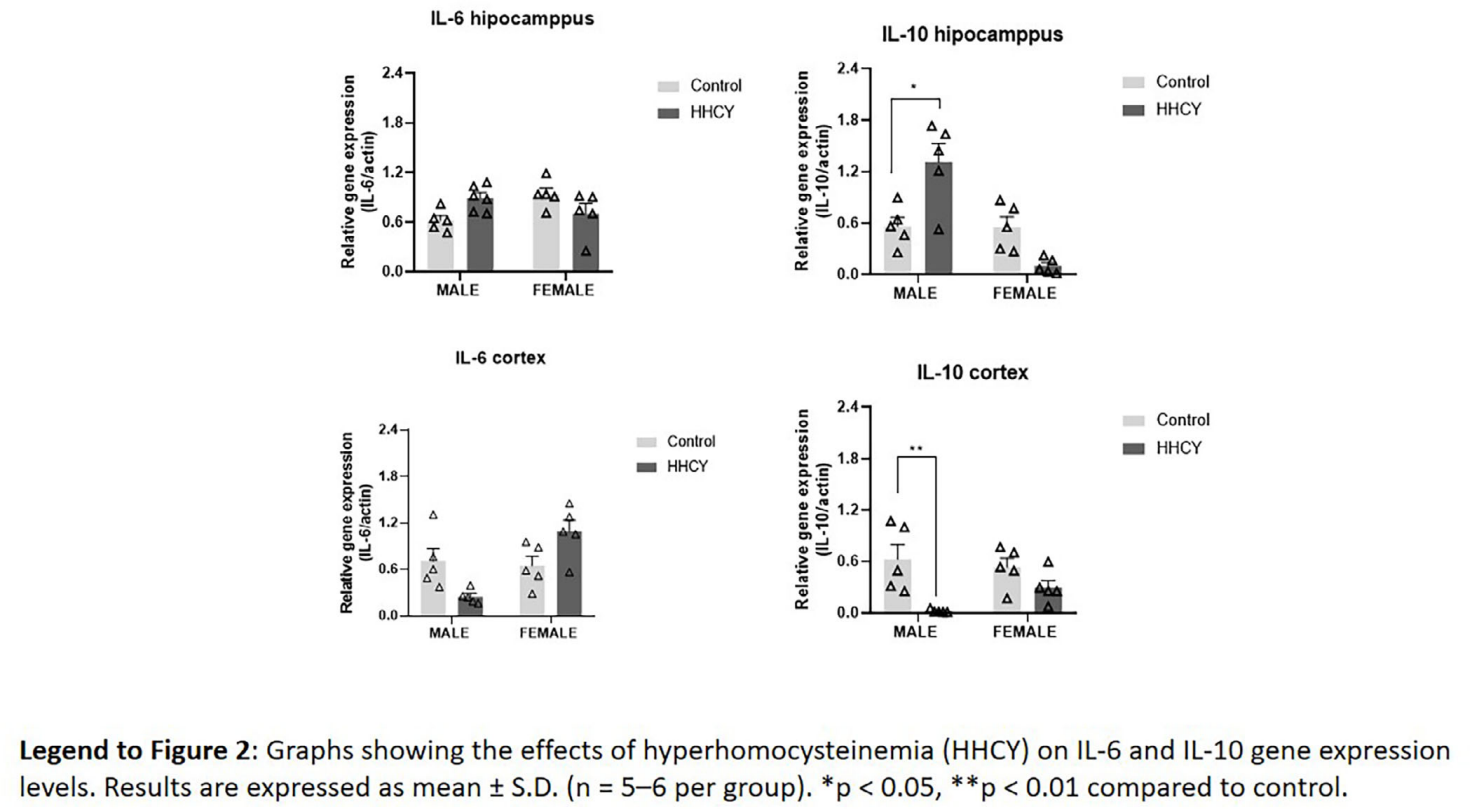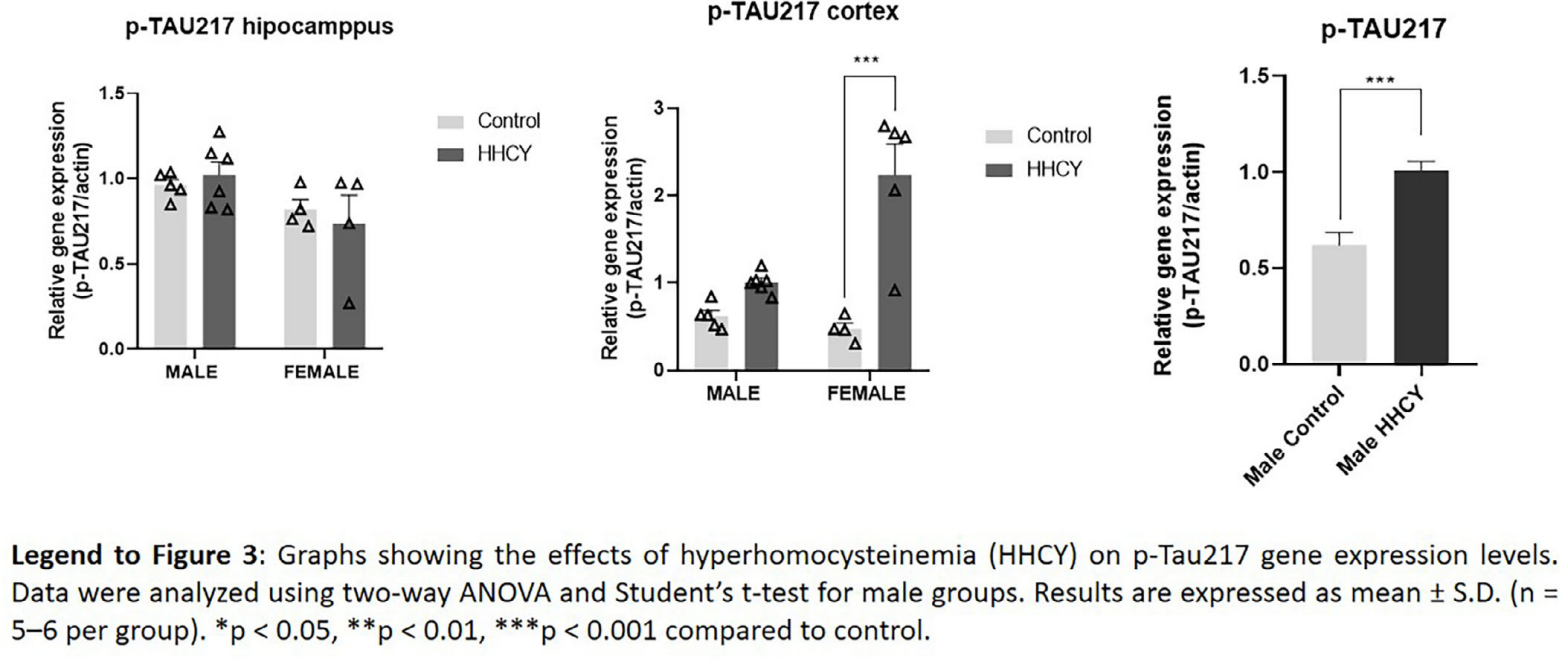# Exploring Homocysteine as a Model for Neurodegenerative Diseases: A Focus on Tau Pathology and Sexual Dimorphism

**DOI:** 10.1002/alz70861_108437

**Published:** 2025-12-23

**Authors:** Alessandra Schmitt Rieder, Laura Teixeira da Rosa, Nicole Soares Lima, Gustavo Ricardo Krupp Prauchner, Victor Camaratta Dossin Bastos, Guilherme Carvalho Serena, Angela Terezinha de Souza Wyse

**Affiliations:** ^1^ Universidade Federal do Rio Grande do Sul, Porto Alegre, Rio Grande do Sul Brazil

## Abstract

**Background:**

Alzheimer’s disease (AD) models play a critical role in understanding the cognitive impairments associated with β‐amyloid and/or tau accumulation. Additionally, sex differences in tau pathology have been linked to disparities in disease progression in clinical settings, which may limit the translational relevance of current models. Homocysteine (HCY) is an sulphur amino acid non proteic that may increase in plasma (hyperhomocysteinemia –HHCY) due to nutritional and genetic factors, and others. HHCY is a risk factor for neurodegenerative diseases, including AD, linked to memory deficits, hippocampal atrophy, and inflammation. Studies associate HHCY with β‐amyloid accumulation, suggesting its potential as an AD model. Therefore, it is relevant to postulate the relationship between HHCY and tau pathology. Thus, we evaluate memory, *p* ‐TAU217 and inflammatory markers.

**Method:**

Wistar rats received subcutaneous HCY from postnatal day 60 to 90. Following treatment, animals underwent the water maze test and were then euthanized for gene expression analysis. Statistical analyses included two‐way ANOVA and Student’s t‐test.

**Result:**

HHCY impaired spatial memory in male rats, as indicated by increased time to find the platform, reduced path efficiency, and worsened learning (*p* <0.05). The hyperhomocysteinemic female rats did not show any of these alterations, although their travelled distance was greater than that of control females (*p* <0.05). Notably, *p* ‐Tau217 expression was elevated in the cortex of female HHCY rats (*p* <0.0001), but not in the hippocampus (*p* >0.05). The HHCY males exhibited a tendency to increase *p* ‐Tau217, confirmed by a Student’s t‐test (*p* >0.05). Interleukin‐10 (IL‐10) levels were altered only in male rats, showing descrese in cortex (*p* >0.05) and enhance in hippocampus (*p* >0.05). The IL‐6 levels were not significantly altered in male and female rats (*p* >0.05).

**Conclusion:**

The results align with clinical observations, showing that tau expression does not correlate with symptom severity across sexes. While male rats displayed memory impairments and interleukin changes typical of HHCY and AD, hyperhomocysteinemic females showed less issues in WM performance but elevated *p* ‐Tau217 levels. These findings reiforce the sex differences in dementia. Thus, since AD models struggle with sex‐specific tau pathology, HHCY could be a useful model for studying tau in females. Supported by INCT (Saúde Cerebral)/CNPq, Brazil.